# Health System Performance Assessment: Does Germany provide good access to healthcare?

**DOI:** 10.1093/eurpub/ckac131.117

**Published:** 2022-10-25

**Authors:** M Blümel, P Hengel, M Achstetter, M Schwarzbach, R Busse

**Affiliations:** Department of Health Care Management, Berlin University of Technology, Berlin, Germany

## Abstract

**Background:**

Providing equal access to health care is a major goal of health systems and a criterion for health system performance assessment (HSPA). The first systematic HSPA for Germany has been piloted in 2021. Access is one dimension of the conceptual framework (others are, e.g., population health, quality, and efficiency), which will be analysed in the following.

**Methods:**

Nine indicators to measure access were selected based on a systematic search of established instruments in (inter)national HSPA initiatives. Included indicators are availability and accessibility of services (e.g., waiting times) and financial risk protection, among others. Other criteria for the inclusion of indicators were data availability and international comparability. Indicators were evaluated in terms of their trend over time (2000-2020), in international comparison (e.g., Austria, Denmark, France), and according to various equity categories (e.g., age, gender, region).

**Results:**

The indicator access to palliative care could not be evaluated due to lack of data. Overall, access is good in Germany. Internationally, Germany performs better than average on most of the indicators, and its performance has improved over time. Physician density in the inpatient and outpatient sectors has increased since 2000 and is above the average of comparator countries. For some specialties, physician density in rural areas is lower than in urban areas, but the gap has decreased in recent years and does not apply to primary care. Furthermore, only 0.3% of the total population report having foregone care, although they had considered it necessary.

**Conclusions:**

Nine indicators were identified and calculated to assess the performance of the German health system in terms of access to healthcare. Access can be assessed as predominantly positive, but inequities exist. Identified gaps and future extensions, e.g., additional data sources, can provide impetus for evidence-based policy management.

**Key messages:**

• First systematic Health System Performance Assessment for Germany has been piloted.

• Access to health care is good in Germany, both over time and in international comparison, but inequities exist.

